# How Clinicians Conceptualize “Actionability” in Genomic Screening

**DOI:** 10.3390/jpm13020290

**Published:** 2023-02-04

**Authors:** Kellie Owens, Pamela Sankar, Dina M. Asfaha

**Affiliations:** 1 Department of Population Health, New York University Grossman School of Medicine, New York, NY 10016, USA; 2 Department of Medical Ethics and Health Policy, University of Pennsylvania Perelman School of Medicine, Philadelphia, PA 19104, USA; 3 Department of Anthropology, University of Pennsylvania, Philadelphia, PA 19104, USA

**Keywords:** actionability, genomic screening, ethics

## Abstract

Over the last decade, the concept of actionability has become a primary framework for assessing whether genetic data is useful and appropriate to return to patients. Despite the popularity of this concept, there is little consensus about what should count as “actionable” information. This is particularly true in population genomic screening, where there is considerable disagreement about what counts as good evidence and which clinical actions are appropriate for which patients. The pathway from scientific evidence to clinical action is not straightforward—it is as much social and political as it is scientific. This research explores the social dynamics shaping the integration of “actionable” genomic data into primary care settings. Based on semi-structured interviews with 35 genetics experts and primary care providers, we find that clinicians vary in how they define and operationalize “actionable” information. There are two main sources of disagreement. First, clinicians differ on the levels and types of evidence required for a result to be actionable, such as when we can be confident that genomic data provides accurate information. Second, there are disagreements about the clinical actions that must be available so that patients can benefit from that information. By highlighting the underlying values and assumptions embedded in discussions of actionability for genomic screening, we provide an empirical basis for building more nuanced policies regarding the actionability of genomic data in terms of population screening in primary care settings.

## 1. Introduction

As advances in genomic technology improve both the utility and cost-effectiveness of genetic testing, many have called for expanded, population-level genomic screening programs [1]. These programs aim to help healthy people to identify genetic predispositions to diseases before they occur or to help tailor drug treatments to their specific needs. However, clinical genomic sequencing produces large amounts of data, much of which is hard to characterize or may have a negligible influence on health. Thus, one of the first questions that these programs face regards actionability: how to assess which types of genomic data have enough evidence and value for the information to be returned to healthy patients [2]. While there are many approaches to answering this question, the concept of actionability has become a primary framework to separate information that may be useful from information that is likely to be irrelevant to patients [3,4,5]. Actionability often refers to the level of evidence regarding the pathogenicity and penetrance of a variant, the efficacy, burden, and availability of interventions, and the severity of potential disease [6]. Even with widely referenced guidelines on actionability from the American College of Medical Genetics and Genomics (ACMG) [7], there is still ongoing debate about how to apply these guidelines to screening programs, and recent research has demonstrated the need for “further demarcation of what exactly constitutes medical actionability” [8].

It is particularly critical to understand the qualitative complexity of actionability in genomic screening because at least 11 genomic screening programs are operating today in the United States alone, with the number of programs expected to expand [9]. These programs offer low-cost or free genetic testing to currently healthy (or unselected) patients, often through primary care providers. Most of these programs offer testing for single-gene disorders, but they vary in their scope and define actionability quite differently. For example, some programs choose to follow guidelines from the CDC Office of Genomics and Precision Public Health, screening patients for only the 10 or 11 genes with the best clinical knowledge base, often referred to as the “CDC Tier One Conditions” [10]. These conditions include Lynch syndrome, hereditary breast and ovarian cancer, and familial hypercholesterolemia. Many others are screening for the 59 genes once included in the ACMG guidance for reporting medically actionable secondary findings (findings unrelated to the original purpose of a genetic test), which includes genes associated with a range of diseases mostly related to cancer or cardiovascular health [11]. This list was updated in 2021 to include 73 genes [7]. A smaller number of programs take a more ad hoc approach to deciding what counts as an actionable result. Finally, some programs offer other types of genetic testing, such as polygenic risk scores for common diseases or pharmacogenomic data on gene/drug interactions. This variation both points to the need for further clarification of what counts as an actionable result and allows for some interesting points of comparison.

Because genomic sequencing produces large amounts of data, it can be difficult to assess what data are both interpretable and valuable in clinical settings. Assessing the value of genomic data also necessitates an understanding of the relationship between that data and a set of potential actions it may set in motion. Many concepts in clinical genomics have been used to theorize this relationship, such as “clinical validity” and “clinical utility.” The National Library of Medicine defines clinical validity as how well the genetic variant being analyzed corresponds to the presence, absence, or risk of a specific disease. Clinical utility refers to whether a test can provide information about the diagnosis, treatment, management, or prevention of a disease that will be useful to a consumer [12]. As our research findings will demonstrate below, actionability often encompasses both clinical validity and utility, combining debates about clinical and laboratory evidence with the value of subsequent clinical actions. Conceptualizations of actionability can also include other dimensions, such as personal utility for patients [13,14].

**Figure 1:**
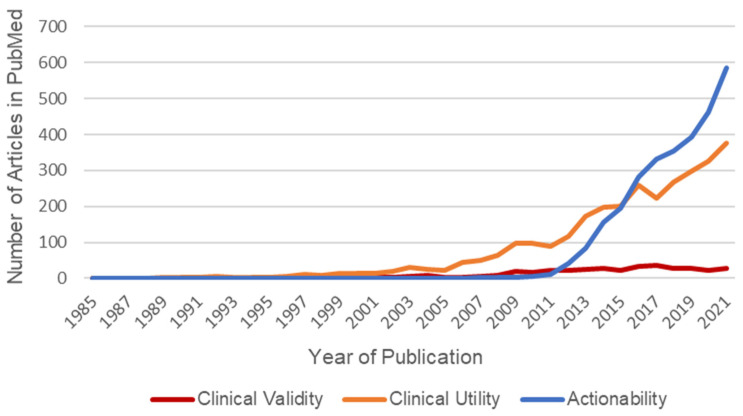
Published genetics articles referencing clinical validity, versus clinical utility, versus actionability. Based on data from three PubMed searches of titles/abstracts: (1) “clinical validity” AND (genetic OR genomic); (2) “clinical utility” AND (genetic OR genomic); (3) (“actionable” OR “actionability”) AND (genetic OR genomic).

Actionability only became a popular phrase in genomics around 2011 but is already more commonly used in the published genetics/genomics literature than clinical validity or clinical utility (Figure 1). Definitions of actionability generally involve three main factors [6]. The first is the level of evidence regarding the pathogenicity and penetrance of a variant. This refers to how confident researchers are that this gene variant is associated with disease, and how likely someone with this gene variant may be to actually exhibit symptoms of the disease. The second is the efficacy, burden, and availability of interventions. For example, if the available intervention for a condition predicted by a genetic predisposition is to remove the stomach, that would be less actionable than a case where someone should be put on mild medication. Finally, actionability generally includes a discussion of the severity of potential disease, where a variant that increased a patient’s risk of something non-life-threatening would be less actionable than something that increased a patient’s risk of sudden cardiac death [15]. The typical example of what many experts in the field would consider *not* being actionable is testing for a genetic predisposition to Alzheimer’s disease, because there are so few treatment options for Alzheimer’s disease [16]. On the other side of the spectrum, genetic testing for predisposition to breast cancer is often considered one of the most actionable types of tests, both because the genetics of breast cancer are better understood than other areas of genetics, and because there is a range of available interventions at different levels of severity for people with a pathogenic variant in a gene such as *BRCA1* or *BRCA2* [10].

Thus, actionability is used to discuss not only which results to return, but what counts as a result in the first place, and what actions, if any, should be taken after receiving results [13]. It is also used to discuss how information can increase or decrease medical uncertainty [17]. Actionability is more than just a concept used in the literature—it is practiced and structures decision-making in a variety of ways, depending on the context [18,19]. Actionability, perhaps obviously, directs attention toward whether genomic information warrants action and reflects its initial development as a strategy to augment diagnosis and treatment in sick patients. As clinical genomic sequencing expands toward healthy or unselected populations for screening in primary care settings, actionability is still widely embraced without there being a consensus regarding its definition and use [15,20]. Prior research has shown that interpretations of actionability are highly variable, relying as much on social context as scientific theory [19,21]. Professional societies, such as the American College of Medical Genetics and Genomics (ACMG) and the European Society for Human Genetics, disagree in their conceptualizations of actionability, and the ACMG has changed the ethical underpinnings of its related guidance on the reporting of secondary findings multiple times (for a more detailed account of these policy changes and disagreements, see Ref. [5]).

Patients also have nuanced, variable understandings of genomic sequencing that do not always match expert definitions of actionability [18,20,22]. Patients are more likely than clinicians to view genomic information as actionable if it could foster lifestyle changes or differences in reproductive decision-making [23,24]. A systematic review found that a significant portion of the lay public requests as much genomic information as possible [23]. However, the question of what counts as genomic “information”, separating signal from noise, is still being debated in the field. Because the majority of research on actionability and the utility of genomic data has focused on patient perspectives, our study instead interrogated the differences between clinical genomics experts and primary care providers.

This article provides a qualitative, empirical examination of how clinicians understand actionability specifically in the context of genomic screening. Based on semi-structured interviews with genetics experts and primary care providers, we find that clinicians vary in how they define and operationalize “actionable” information. Two main types of variation emerged from our analysis: (1) disagreement about both the quality and quantity of evidence required; (2) differences when identifying which “actions” should or must be available to patients. Variations in clinicians’ perspectives about evidence often hinged on the type of genetic test being offered and the clinician’s area of expertise. For example, many primary care providers discussed a need for population-level clinical trials, while genomics experts felt more comfortable with other types of clinical evidence of utility. Variations related to the available actions often centered on concerns about the capacity of health systems or an assessment of market incentives. For example, some health systems limited their ambitions regarding genomic screening due to their restricted capacity to offer follow-up care, while others sought to expand access to services such as polygenic risk scores, due to a perceived market and potential profitability. These findings suggest both that there are critical differences in clinicians’ expectations of evidence for genomic screening programs and also that discussions about available “actions” center on concerns about capacity or marketability rather than clinician decision-making. As genomic screening programs become more popular, it will be critical to understand these tensions surrounding actionability and address them in the design and implementation of such programs.

## 2. Materials and Methods

This is a qualitative study based on semi-structured, in-depth interviews with primary care providers and genetics experts. Our methods are reported below, using the consolidated criteria for reporting qualitative research (COREQ) guidelines [25] (for full details, see Supplementary File S1). This research was deemed exempt by the Institutional Review Board at The University of Pennsylvania.

### 2.1. Participant Recruitment

We used purposive and snowball sampling to recruit participants for this study. We recruited participants of three types: (1) primary care providers; (2) genetics experts (which we defined as clinical geneticists, clinical laboratory geneticists, or genetic counselors); (3) clinicians with expertise in both genetics and primary care. We chose these specialties so that we could assess the similarities and differences between the types of clinicians most likely to be involved in population genomic screening programs. We recruited participants via email and followed up one to two times by email, as necessary. We contacted 64 potential participants. Of these, 35 participants completed interviews, while 29 potential participants were unresponsive or unavailable. Of those who completed interviews, 20 participants were genetics experts, 10 participants were primary care providers, and 5 participants had expertise in both genetics and primary care; 22 participants worked in health systems that offered genomic screening programs, while 13 worked in environments that did not currently offer genomic screening. Participants gave verbal consent before beginning the interview and were offered USD 50 gift cards for their assistance in the research.

### 2.2. Data Collection

We developed an in-depth, semi-structured interview guide (Supplementary File S2), based on our experiences in the field and a review of the relevant literature. We revised the guide after internal pilot testing and allowed participants to direct the conversations within the bounds of the guide. We asked participants about their professional training and daily work, their general views on population screening, their understanding of actionability, and their thoughts on related ethical issues such as equity and privacy in genomics. Kellie Owens conducted all interviews virtually, via Zoom video conferencing software, between January 2021 and July 2022, including writing field notes during or after interviews. Interviews lasted approximately 30–60 min, and audio recordings of the interviews were professionally transcribed. Interviews with each participant type continued until we reached thematic saturation.

### 2.3. Data Analysis

We analyzed the interview transcripts and field notes, following the principles of grounded theory [26] and situational analysis [27]. This is an iterative process that allows themes, concepts, and theoretical insights to arise from the data, paying particular attention to differences between groups and positions not being taken. We developed and continually revised a codebook, then used NVivo for Mac 1.7.1 software for coding and thematic analysis. Kellie Owens and Dina M. Asfaha coded the interview transcripts, then any discrepancies were resolved via discussion. Data analysis occurred from January to September 2022.

## 3. Results

The following sections will first address the rise of “actionability” as a key framework used to assess the utility of returning a genetic/genomic test result to a patient and explore variations in how clinicians conceptualize this phrase. We will then discuss the main findings of our analysis, which focus on the ways in which actionability is differentially considered and practiced in context. We conclude with a discussion of the impacts of this research on the future of genomic screening programs and on patient care.

### 3.1. Definitions of Actionability

We asked participants to define “actionable” in their own terms, specific to the context of population screening. Some participants discussed well-defined criteria and others took a more amorphous, “I know it when I see it”, approach. Those with specific ideas of actionability offered a range of responses, from restrictive to expansive. For example, a participant on the restrictive side of the spectrum suggested:

“[My definition of] actionable would have to be a truly pathogenic change, for which there is a known treatment or surveillance recommendation that impacts a lifetime of medical management and saves lives.”

—Clinical geneticist

This participant considered actionability to refer to those interventions that require serious changes in medical management and a demonstrated ability to improve patient outcomes. Others had a more expansive view:

“I think of [actionability] really broadly: anything that has the potential, either now or in the future, to modify either life choices or medical treatment … We [could] say, ‘Oh, is that actionable? There’s no treatment for it.’ But then [patients] can enroll in clinical trials and things like that … And I think it’s important to consider the social and emotional aspects of what we consider actionable, too, because it’s very personal. So, some things that someone else wouldn’t consider actionable would be really important psychologically and … wouldn’t necessarily be, ‘Oh, we’re gonna change this medication,’ or do a surgery, or things like that.”

—Clinical geneticist

Thus, despite published guidelines and metrics for actionability, we find little evidence of consensus among clinicians in practice, especially in the context of population screening. At the same time, while documenting these differences in how clinicians define actionability is important, the more informative parts of our interviews focused on the context and actual practice of declaring a gene/disease pair as being actionable or disclosing a result to patients. The following sections outline our main findings on how “actionable” results are differentially considered and practiced in context.

### 3.2. Evidence Is Contextual

#### 3.2.1. The Question of Now vs. Later

One of the key components of actionability is an assessment regarding what types of evidence, and how much of that evidence, are required before a result should be returned to patients. This is not a straightforward question, and our discussions with participants brought up a range of factors related to the context of genomic screening that were important to different people. First, participants had different ways of balancing the tension between providing potentially life-altering data and care to patients as quickly as possible, versus waiting for more and better evidence of validity or utility. For example, some clinicians, driven by the principle of “do no harm”, were primarily concerned about the risks of overtreatment based on uncertain science:

“This is how close we were to really making a big mistake. We looked at all this information, you know, we used the best databases available. We used ClinVar. We used ClinGen. We [had another laboratory perform] a separate assessment of the variants before they returned the results to us. We would look at everything and we would say, ‘Okay, these patients look like they might have a pathogenic or likely pathogenic change.’ [At the last minute, we learned that another laboratory] had actually revised their classification from pathogenic to VUS [variant of uncertain significance]. And so we [did not] return this variant, but it was pretty close to us actually coming to that point. And it actually happened also with a couple of the [heart condition] genes as well, early on, that variants that we thought were likely pathogenic turned out not to be. And so that’s a really big concern, that you’re overtreating not because … you know, it was even the best standard at that time, I guess, but the standard had moved.”

—Clinical geneticist

This clinician was worried that genetic screening was moving too quickly from research into clinical practice, without a solid evidence base at the population level. He cautioned that the balance of risks and benefits of genetic testing is different for someone with a family history or phenotype of disease than for an unselected, otherwise healthy patient. Many clinicians echoed this approach, preferring to wait to deliver information to patients until they could have greater certainty in its value.

Still, other clinicians spent more of our discussion focused on the benefits that they could be offering patients immediately, even while recognizing that the science of genomic screening will continue to evolve. These clinicians either did not see the value in waiting for additional evidence or thought that waiting was unrealistic:

“We have this weird middle line of, like: this [more evidence] is the ideal, [but] this is reality. We don’t want to leave patients hanging, because, like, the reality happens before the ideal does.”

—Genetic counselor

While this clinician would ideally prefer to have better evidence for the utility of genomic screening before implementing these programs, she sees more value in offering this service to patients now. Often, clinicians would discuss the dangers of waiting for better evidence in quite personal terms:

“Sometimes the ultimate question is, ‘Think about yourself and your family and would you want to know? Would you pursue this testing if it were offered to you?’ And I think many people would say, ‘Yes.’ And then, the argument is … Isn’t this something that we should be offering for our patients now because it can have a major effect for them?”

—Primary care provider

It is not surprising that participants made different calculations of how to weigh potential risks and benefits, as we see this across health and social domains. Perhaps most dramatically, the COVID-19 pandemic brought to light how people vary in their propensity for action in the face of uncertain evidence, fueling highly politicized debates about prescribing drugs such as hydroxychloroquine and remdesivir to treat COVID-19 symptoms.

Where participants’ views fell along this spectrum tended to match the attitudes of others at their institution. While the methods of the current study do not allow us to explain why participants in the same institution often espoused the same balance between evidence and action, theories from other social scientists would suggest that commitment to a position is a consequence rather than a cause of affiliation [28]. In the context of clinical genomics, we would hypothesize that participants develop their views about actionability within their workplaces, rather than choosing to work in a place that supports their own already-developed views.

To summarize, the question of whether to offer genomic screening now or later was at the forefront of their minds for our participants when discussing actionability. We observed differences in how participants thought through the value of being able to help some people immediately (while also giving others potentially incorrect information) versus waiting until the science of genomic screening was more settled, to avoid any potential harm from incorrect interpretations. Importantly, weighing these potential benefits and harms will always be a matter of values, and the matter cannot be solved by simply “following the science”. Genomic screening programs will need to take into account these values and choices in their design and implementation.

#### 3.2.2. What Evidence Matters?

In our sample, the participants’ professional identities seemed to impact which types of evidence they deemed relevant to discussions of actionability. Participants with genetics expertise who expressed concerns about evidence were generally most concerned about the evidence of accuracy regarding a particular result, such as whether a variant was correctly classified. Many participants with genetics expertise reported investing significant effort to check and potentially even reclassify gene variants, based on their own expertise and a particular patient phenotype, matching recent research showing that this is a common practice among genetic counselors [29]. Participants with genetics expertise also reported less trust in genetic lab reports:

“I struggle a little bit with the fact that all labs are using the ACMG criteria but yet we can still get different answers from labs even when they’re using the same criteria.”

—Genetic counselor

“This is probably the biggest struggle that we have currently in the field. If I really were to go through and rank, like, what are all of my frustrations, [laboratory variability] is probably top of the list right now because there’s not a central place to collect [info about laboratories] and be able to compare back to who’s giving better reports, who’s using actual ACMG classification criteria, who’s doing whatever with whichever lab results come back.”

—Genetic counselor

In contrast, primary care physicians expressed much less concern about variant classification or the accuracy of a specific result:

“I don’t disagree with the genetic lab results. I wouldn’t know how to disagree with genetic lab results.”

—Primary care provider

Instead, primary care providers paid greater attention to population-level data showing the efficacy of genetic screening programs. They wanted to see randomized controlled trials on the efficacy of population genomic screening, along with professional society guidelines.

“There’s a high bar of evidence that’s required before some big new [screening] initiative is going to be put in place. You’ll hear a lot about barriers, like, ‘Oh, our EHR isn’t ready, doctors don’t know how to do this, they don’t know how to talk to patients about it.’ All of that’s true, but all of that would fall into place pretty quickly if [a] clinical guideline from USPSTF [the U.S. Preventive Services Task Force] says, like, ‘This should be done.’ And why haven’t they said that? Maybe they haven’t asked, but I think they also know that the evidence just isn’t there, and, you know … USPSTF would have a pretty high bar of evidence that would typically rely on RCT-level evidence. So, I mean, that’s the big one … If that happens, everything else falls into place.”

—Primary care provider with genetics expertise

For some of these primary care providers, the lack of clinical trial evidence for more expansive screening programs made them reluctant to add genomic screening to their practice. Alternatively, they preferred to screen for those conditions with the most population-level evidence, such as conditions classified as CDC Tier One.

Differences in perspective between genetics experts and primary care providers may also be due to the perceived tension between the tenets and realities of precision medicine versus evidence-based medicine. Below, a family physician with training in genetics explained how she understands the evidentiary standards of the two fields:

“So, precision medicine is the n-of-1, right? Family medicine has been trained, evidence-based medicine has been stuffed down their throat, right? Since day one … To reach the level of what’s considered evidence-based medicine, you have to have a significant N, and that’s not what precision medicine is. And that scares them because this is where they’ve trained, this is where their comfort level is. They can go to their references, they can go to their data, they can go to the national guidelines. But precision medicine isn’t that … Precision medicine is pragmatic, right? You can’t do a huge retrospective or prospective randomized control trial, it just, it doesn’t work. And so, it’s a new comfort zone. So, while we’re educating these young docs to have the competencies, they also need to have the confidence to practice n-of-1 medicine.”

—Primary care provider with genetics expertise

Other participants highlighted the importance of other, “softer”, types of evidence in their practice:

“I don’t think everything needs a randomized control trial. It always reminds me of a commentary that was published in a journal … talking about how you don’t need a randomized control trial to prove that parachutes work when you skydive. And so I think pharmacogenomics is kind of like that. And I think there’s a lot of soft outcomes too. I see in my patients personally, sometimes just them getting tested and knowing that we’re aware of their results makes them more open to trying a new medication or to give something a longer shot … And sometimes that will help them have a better outcome, not necessarily because the test led us to that but the act of testing helped them out.”

—Primary care provider with genetics expertise

Because evidence generation in clinical genetics tends to appear different from evidence generation in primary care, we expect to see continued disagreements about both the quality and quantity of evidence required for genomic data to be actionable in the context of population screening. Depending on professional identity, participants highlighted either the importance of generating evidence to support the proper classification of individual gene variants or the importance of collecting clinical trial data regarding population-level outcomes.

#### 3.2.3. Required Evidence Varies by Test Type

Debates about evidence and actionability also depended heavily on the type of genomic screening under question. There are three main types of screening programs: screening for single-gene disorders, screening for complex disorders via polygenic risk scores, and pharmacogenomic screening. Genetic testing for single-gene disorders is the most common, and clinicians often have a good understanding of the underlying biological mechanisms that cause disease. As a genetic counselor explains:

“With Mendelian [single-gene] testing, we are very grounded in an understanding of biological mechanisms … So the way that we demonstrate utility is we first establish this gene has this role and pathway in disease. We can know what [specific variants] people have; we have to, to some extent, do a discovery type of approach for each individual, and then decide if [that variant] is pathogenic in this gene that we know. So you prove utility on an individual basis almost but it’s in the context of, like, we know this disease mechanism. In a lot of medicine, those mechanisms are not as rigorously known in advance, right? So that’s worked for us, and we’ve had a lot of loopholes, I think, in how we were able to approach [evidence generation] because there’s a lot of rare diseases … or, like, just genetics, in general, has been very different from the rest of medicine.”

—Genetic Counselor

Collecting population-level outcome data is also, admittedly, difficult and time-consuming. A clinician describes this process for pharmacogenomic testing:

“One of the criticisms of pharmacogenomics is that we’re still working on growing the evidence of outcomes data. Whenever you’re on … medication, collecting the outcome data whenever you’ve used pharmacogenomics to guide the therapies is probably not that easy. Because you have to follow people so far … to see if the outcome actually occurred or not. That’s, I think, what payers and health plans are looking for. And there is some evidence that exists for that. But it’s not totally, you know, great evidence. And so I think that’s the hardest thing.”

—Primary care provider with genetics expertise

Other types of genetic testing, such as polygenic scores for complex disorders, do not have as much evidence of the underlying biological mechanisms. Instead, polygenic scores are created by comparing the genomes of individuals with and without a particular disease. Thus, the genetic counselor above explains how she sees the differences in the required types of evidence between these two forms of tests:

“[For] polygenic scores … I can completely empathize with the norm that there should be randomized controlled trials. People that do randomized controlled trials are not geneticists, [geneticists] don’t know how to do that. They don’t know how to do, like, a big epi [epidemiological] study … So it’s this tension of geneticists taking the same approach of, ‘Oh, we’ve proven a relationship and that’s enough to now test people.’ And it’s like, no, it’s an association. It’s not grounded in biology. It’s a very different bit of information.”

—Genetic counselor

This perspective suggests that different types of genetic tests could require different approaches regarding evidence generation and that it would be a mistake not to differentiate between them. This, in turn, could affect how clinicians and researchers understand the actionability of these tests.

### 3.3. Actionability and Institutional Capacity

In addition to questions about scientific and clinical evidence, actionability also requires thought about the types of actions available to patients, clinicians, or health systems. What can patients and providers actually do with the information presented to them? If they cannot do anything, is that data worth returning? Before completing our interviews, we expected to have discussions with participants about the specific clinical decisions they make and the actions that they take based on genomic screening results. Instead, when we asked participants about how they understand the clinical actions that should or should not be taken based on genomic data, their answers were more general and focused primarily on the capacity of health systems.

Specifically, the lack of institutional capacity to fully integrate population genomic screening was a primary concern for many participants. Participant perspectives varied as to why capacity was limited. Some thought that there were so many other, more immediate, population health problems to tackle that genomics just did not make the cut:

“Right now, I can understand why for many [primary care providers], you know, they’re dealing with people who have COVID, they’re dealing with the flu, they’re dealing with people who … have diabetes, and so forth, and fundamentally, they wanna know if I ordered these [genetic tests], how are they gonna impact what I’m prescribing, or managing? And right now, in many cases, it doesn’t impact them.”

—Genetic counselor

Others focused on the lack of genetics training for providers, making it difficult for those providers to know how to interpret and address the findings of a genetic test:

“I was at a clinic talking to primary care providers … And a doctor was sitting next to me and he said, ‘I won’t talk to my patients about genetics. I’m not comfortable…’ And so you’re constantly playing catch up … because the clinicians are overworked, they’re undertrained, frankly, and they don’t have the infrastructure to deal with [the question of] what is the clinical decision support tool necessary for 18- to 35-year-old women with *BRCA1* and *2*?”

—Clinical geneticist

Concerns about capacity were sometimes the primary drivers of decision-making around actionability, rather than evidence. For example, a clinician describes how his health system chose to screen for a limited number of conditions:

“And I think the smart decision we made at the time was that because we’re a safety net hospital, [with] almost no research infrastructure … no genetics department … we would return results on the CDC Tier 1 conditions. And I think that decision was probably the right one. And, subsequently, many other health systems are recognizing that, you know, the ACMG’s guidelines on incidental findings … [are] not meant for all-comers, you know, for moderate risk population screening. And that, still, the CDC Tier 1 conditions are probably appropriate to return to all-comers.”

—Clinical geneticist

This clinician first mentions a lack of infrastructure, before also commenting on the higher levels of evidence for CDC Tier 1 conditions in comparison to other, more expansive lists of genes. He continued to describe his discomfort with expanding their testing because having more patients with pathogenic results would not necessarily correspond to those patients getting the care they needed:

“To get to imagining returning 4% pathogenic, likely pathogenic, results in [an expanded list of genes] would be impossible. And so you’d have all these indications in the medical record and you’d have folks who were not getting the standard of care, given those indications. And so the question is, do you then interpret them and just know that?”

—Clinical geneticist

Concerns about capacity may vary depending on the level of resources at the health system where a participant worked, at least in our sample. For example, participants from health systems with more resources did not discuss problems related to maintaining standards of care, and instead discussed developing more expansive genomic screening that could eventually benefit a far wider number of people:

“[Our hospital has] extra money sometimes to reinvest in developing new services that might not have a clear guideline behind them or clearly established utility … things we think will be important one day, and we’re gonna back it. So they decided they wanted to build more preventive genetics, population screening infrastructure in a more robust way, and really latched on to both polygenic risk scores and pharmacogenomics since the target audience for those are bigger. It’s more of a general population approach.”

—Genetic counselor

This participant did not report any concerns about health system capacity—instead, her health system was working to expand testing to the largest number of patients. As genomic screening programs become more popular, we may see differences in whether health systems have the available infrastructure, both to offer testing to more patients and to support the patients that do undergo testing and need follow-up care.

Generally, our responses from participants suggest that they are less interested in debating what types of specific clinical actions make a genetic result worth returning to patients and are more concerned about ensuring follow-up care for even the most straightforward screening efforts. For example, we spent less time discussing whether a genetic predisposition to Alzheimer’s disease was actionable, given the lack of clinical actions available. Instead, participants highlighted their efforts, and often frustrations, regarding providing patients with resources, even for CDC Tier One conditions with the clearest practice guidelines.

## 4. Discussion

In this qualitative study of clinician perspectives on the actionability of genomic data for population screening, we find that clinicians vary in how they define actionability and how they utilize the concept in practice. First, clinicians are often unable to articulate what counts as actionable information, but suggest that they “know it when they see it.” Even when participants were able to define actionability, their responses varied from restrictive to expansive. In our analysis of how clinicians implement the concept of actionability in practice, we make two main contributions to the literature on precision public health: (1) debates about the quantity and quality of evidence required for actionability are contextual, and are driven by value judgments; (2) clinicians perceive capacity and infrastructure to be major barriers to implementing genomic screening programs, and worry that this could lead to real differences in follow-up care between health systems with different levels of resources. Importantly, however, some participants argued that infrastructure to support genomic screening will “fall into place” if better evidence of clinical utility is generated. Thus, we find that the dominant concern for clinicians regarding actionability was related to standards of evidence, rather than clinical actions.

As a small-scale qualitative study, our methodology and data face several limitations. First, we cannot provide evidence of how our participants’ views align with their peers across the country, because our sample is not designed to be representative of the population. Second, by focusing our interviews on genetics experts and primary care providers, we excluded the perspectives of other relevant stakeholders, such as patients, funders, and regulators. Still, our findings suggest clear disagreement and variation among clinicians who serve as key gatekeepers to population screening. Additionally, due to the depth of our interviews, this study provides greater clarity on the underlying values and assumptions that drive differences in opinion on actionability.

As more population genomic screening programs develop across the United States, we encourage the designers and implementers of these programs to address these value judgments head-on, in order to develop programs that provide the ideal balance of risks and benefits, as defined by their local context. Different communities, for example, may place a different weight on the value of intervening in the moment versus waiting for better clinical evidence. This may be particularly true for rare diseases, where large-scale clinical evidence is hard to generate. While professional societies such as the ACMG provide guidance on the types of evidence and actions required to move forward with population screening [30], we suspect that there will never be a universally applicable “correct” way to define actionability for population screening. Instead, genomic screening programs should develop transparent, community-engaged processes to weigh these values and assumptions as they design and implement their programs.

## Data Availability

The data presented in this study are available on request from the corresponding author. The data are not publicly available to help protect the privacy and confidentiality of research participants.

## Supplementary Materials

The following supporting information can be downloaded at: https://www.mdpi.com/article/10.3390/jpm13020290/s1, File S1: Consolidated criteria for reporting qualitative studies (COREQ); File S2: Interview Guide.
