# Supplementary material for: How Clinicians Conceptualize “Actionability” in Genomic Screening
Source: J Pers Med. 2023 Feb 4;13(2):290. doi: 10.3390/jpm13020290 (PMC9959215; doi:10.3390/jpm13020290)
Supplement: Supplementary file 1 [file jpm-13-00290-s001.zip › Actionability Supplementary Files/Actionability_COREQ_Checklist.pdf]

Supplemental Materials S1

Consolidated criteria for reporting qualitative studies (COREQ): 32-item checklist

Derived from: Tong, A.; Sainsbury, P.; Craig, J. Consolidated criteria for reporting qualitative research (COREQ): a 32-item checklist for interviews and focus groups. *Int J Qual Health Care* **2007**, *19*, 349-357, doi:10.1093/intqhc/mzm042.

No. Item	Guide questions/description	This Research
**Domain 1: Research team and reflexivity**		
Personal Characteristics		
1. Interviewer/ facilitator	Which author/s conducted the interview or focus group?	*Owens*
2. Credentials	What were the researcher's credentials? E.g. PhD, MD	*PhD, MSME (Owens), PhD (Sankar), MA (Asfaha).*
3. Occupation	What was their occupation at the time of the study?	*Postdoctoral Fellow/Assistant Professor of Medical Ethics (Owens), Associate Professor of Medical Ethics (Sankar), Doctoral Candidate in Anthropology (Asfaha)*
4. Gender	Was the researcher male or female?	*All female*
5. Experience and training	What experience or training did the researcher have?	*Dr. Owens is a sociologist and medical ethicist with expertise in qualitative research and in-depth interviewing. Dr. Sankar is a medical ethicist and leading scholar of the ethical, legal, and social implications of genetics/genomics. Ms. Asfaha is a Doctoral Candidate in Anthropology with expertise in ethnographic methods.*
Relationship with participants		
6. Relationship established	Was a relationship established prior to study commencement?	*No prior relationship.*
7. Participant knowledge of the interviewer	What did the participants know about the researcher? e.g. personal goals, reasons for doing the research	*Professional background, research interests, reasons for doing this research.*
8. Interviewer characteristics	What characteristics were reported about the interviewer/facilitator? e.g. Bias, assumptions, reasons and interests in the research topic	*Owens' training as a sociologist lends her a critical analytical toolkit, and her identity as a white, cis-het woman likely*

		influences participant interactions.
**Domain 2: study design**		
Theoretical framework		
9. Methodological orientation and Theory	What methodological orientation was stated to underpin the study? e.g. grounded theory, discourse analysis, ethnography, phenomenology, content analysis	*This study uses the principles of grounded theory [21] and situational analysis [22].*
Participant selection		
10. Sampling	How were participants selected? e.g. purposive, convenience, consecutive, snowball	*Purposive and snowball sampling*
11. Method of approach	How were participants approached? e.g. face-to-face, telephone, mail, email	*Contacted by e-mail by Dr. Owens*
12. Sample size	How many participants were in the study?	*35*
13. Non-participation	How many people refused to participate or dropped out? Reasons?	*29 people did not respond to email requests or declined an interview. No one dropped out after beginning participation.*
Setting		
14. Setting of data collection	Where was the data collected? e.g. home, clinic, workplace	*At participants' homes or in their offices, online via Zoom.*
15. Presence of non-participants	Was anyone else present besides the participants and researchers?	*No*
16. Description of sample	What are the important characteristics of the sample? e.g. demographic data, date	*10 participants were primary care providers, 20 participants were clinical geneticists or genetic counselors, and 5 participants had expertise in both primary care and genetics. Participant demographics: Gender: 24 Women, 7 Men, 4 Not Reported. Race: 22 White, 5 Asian, 3 More than One Race, 5 Not Reported. Interviews were conducted between 2021-2022.*
Data collection		
. 17. Interview guide	Were questions, prompts, guides provided by the authors? Was it pilot tested?	*Dr. Owens and Dr. Sankar developed the interview guide, provided in the supplemental material S2. The interview guide was pilot tested internally with*

		Dr. Owens' colleagues, and revised accordingly.
18. Repeat interviews	Were repeat interviews carried out? If yes, how many?	*No*
19. Audio/visual recording	Did the research use audio or visual recording to collect the data?	*Audio and video recording*
20. Field notes	Were field notes made during and/or after the interview or focus group?	*Yes*
21. Duration	What was the duration of the inter views or focus group?	*Approximately 30 minutes to 1 hour.*
22. Data saturation	Was data saturation discussed?	*Yes, interviews stopped once data saturation was reached.*
23. Transcripts returned	Were transcripts returned to participants for comment and/or correction?	*No*
**Domain 3: analysis and findings**		
Data analysis		
24. Number of data coders	How many data coders coded the data?	*Two (Owens and Asfaha)*
25. Description of the coding tree	Did authors provide a description of the coding tree?	*No*
26. Derivation of themes	Were themes identified in advance or derived from the data?	*Both*
27. Software	What software, if applicable, was used to manage the data?	*NVivo*
28. Participant checking	Did participants provide feedback on the findings?	*No*
Reporting		
29. Quotations presented	Were participant quotations presented to illustrate the themes/findings? Was each quotation identified? e.g. participant number	*Participant quotations are included and identified only by professional training, to preserve anonymity.*
30. Data and findings consistent	Was there consistency between the data presented and the findings?	*Yes*
31. Clarity of major themes	Were major themes clearly presented in the findings?	*Yes*
32. Clarity of minor themes	Is there a description of diverse cases or discussion of minor themes?	*Yes*
