# Supplementary material for: How Clinicians Conceptualize “Actionability” in Genomic Screening
Source: J Pers Med. 2023 Feb 4;13(2):290. doi: 10.3390/jpm13020290 (PMC9959215; doi:10.3390/jpm13020290)
Supplement: Supplementary file 1 [file jpm-13-00290-s001.zip › Actionability Supplementary Files/Actionability_Interview_Guide_Final.pdf]

Supplemental Materials S2

Interview Guide: Conceptualizing Actionability in Clinical Genomic Screening

This guide provides a list of sample questions that will be asked during the semi-structured interviews. The list is not exhaustive, and the questions will be tailored to fit the conversation.

Introduction:

- How would you describe your medical specialty and interests?
- What does a typical work week look like for you?
- How often do you order genetic/genomic testing for a patient?
    - Probe as relevant (how many times per week/month/year)
- What kind of training have you received regarding genetic/genomic testing?
    - What do you wish you knew more about in regards to genetic/genomic testing?

Attitudes Regarding Genomic Screening:

- What excites you about genomic screening for the general population?
- What makes you nervous about genomic screening for the general population?
- How do you feel about implementing whole genome or exome sequencing as routine medical care?
- What do you think the role of genetic and genomic testing should be in primary care settings?
- What kinds of information do you need to determine whether genomic screening would be appropriate or inappropriate for a patient?
    - Probe: What makes someone a good candidate?
    - Probe: What makes someone a bad candidate?
- What kinds of information do you need to determine whether any kind of screening test (such as cancer screening) is appropriate for a patient?
- How would you define "actionable" in clinical genomic screening?
    - Can you give me a specific example of something you would consider actionable? Why?
    - Can you give ma specific example of something you would consider not actionable? Why?
- Genomic screening is likely to be less accurate for non-European ancestry populations. Can you talk about whether or how that influences your views about genomic screening?
    - Does this impact your conversations with patients?
- How do you think genomic screening could affect equity in healthcare?
- How do you think about privacy in the context of genomic screening?
- For clinicians ordering genomic screening tests:
    - How do you discuss the decision to undergo genomic screening with patients?
        - What do you think patients often misunderstand about genomic screening?
    - Please share a few examples of when genomic screening has been useful for a patient.
    - Please share a few examples of when genomic screening has not been useful for a patient.
    - Please share an example of when you were unsure about how to interpret a finding, if applicable.
    - When you receive test results from a laboratory, what processes do you follow before you return those results to a patient?

Closing:

- We are studying the ways that clinicians understand the utility and actionability of genomic screening. What else do you think I should have asked you about this topic?
- Is there anything else you would like to add?
